# Prevalence, seasonality, and antimicrobial resistance of thermotolerant *Campylobacter* isolated from broiler farms and slaughterhouses in East Algeria

**DOI:** 10.14202/vetworld.2020.1221-1228

**Published:** 2020-06-28

**Authors:** Mohamed Baali, Mohamed Lounis, Hanan Laidouci Al Amir, Ammar Ayachi, Ahcen Hakem, Ahmed Kassah-Laouar

**Affiliations:** 1 Laboratory of Food Hygiene and Quality Insurance System, High National Veterinary School, Rue Issad Abbes, Oued Smar, Algiers, Algeria; 2 Department of Agroveterinary Science, Faculty of Natural and Life Sciences, University of Ziane Achour, Road of Moudjbara, Djelfa, Algeria; 3 Department of Food and Water Bacteriology, Pasteur Institute of Algeria, Road of Petit Staouéli, Dely Brahim, Algiers, Algeria; 4 Biotechnology Laboratory of Bioactive Molecules and Cellular Physiopathology, Faculty of Natural and Life Sciences, University of Batna 2, Batna, Algeria; 5 Center of Agro-pastoralism, Djelfa, Algeria; 6 Central Laboratory of Medical Biology, Anti-Cancer Center, Batna, Algeria

**Keywords:** antimicrobial resistance, broilers, poultry farms, slaughterhouses, thermotolerant *Campylobacter*

## Abstract

**Aim:**

The current study was carried out to determine the prevalence, seasonality, and antimicrobial profile of thermotolerant *Campylobacter* isolated from broiler chickens in Batna, East Algeria, from June 2016 to June 2018.

**Materials and Methods:**

A total of 960 samples, including 480 cloacal swabs, 240 cecal contents, and 240 neck skin samples collected from 6 poultry farms and 12 slaughterhouses, were included in this study. After isolation and identification, susceptibility to seven antimicrobial agents was tested by the disk diffusion method. The seasonality of *Campylobacter* infection at broiler farms was statistically analyzed.

**Results:**

The data showed that 65%, 55%, and 70% of the cloacal swab, neck skin, and cecal content samples were contaminated with thermotolerant *Campylobacter* strains, respectively (p<0.05). Among the isolated campylobacteria, *Campylobacter jejuni* was the predominant species (73.5%). Sampling season exhibited a significant impact on the prevalence of *Campylobacter* (p<0.01), with peak occurrence in summer. All of the isolates were susceptible to gentamicin and resistant to ampicillin and amoxicillin/clavulanic acid, while 83.3% of them were resistant to erythromycin. Interestingly, 16 different resistance profiles were noted, with the combination of “ampicillin, amoxicillin/clavulanic acid, chloramphenicol, erythromycin, and tetracycline” being the most common, identified in 20.7% of isolated strains.

**Conclusion:**

This study demonstrates the presence of a high contamination rate of multidrug-resistant *Campylobacter* in farms and slaughterhouses in East Algeria. These findings underscore the need to apply strict control measures to avoid any associated public health hazard among Algerian consumers. This initial finding of the contamination of poultry with this zoonotic pathogen in East Algeria suggests the value of periodic comprehensive evaluation of associated disease in poultry as well as in humans in this region.

## Introduction

*Campylobacter* species, *Campylobacter jejuni*, *Campylobacter coli*, *Campylobacter lari*, and *Campylobacter upsaliensis*, are principally foodborne zoonotic pathogens frequently isolated from a variety of animal species such as poultry and cattle [1]. These pathogens threaten public health globally and are considered the most common bacteria inducing gastroenteritis in humans. They can be fatal in very young children and immunosuppressed patients. The majority of campylobacteriosis cases are caused by *C. jejuni* and *C. coli*, accounting for 90% and 5-10% of cases, respectively [2].

Among animals eaten by humans, poultry is a major source of human campylobacteriosis, where both the handling and the consumption of improperly cooked poultry meat were identified as major risk factors for infection [3]. Poultry usually becomes infected at farms; however, little is known about the sources of infection and possible predisposing factors. In addition, cross-contamination of chicken carcasses with *Campylobacter* was documented mainly at the scalding and evisceration stages [4]. This represents a public health concern to poultry consumers. Compounding the risk further is the increasing resistance to fluoroquinolones, the most common treatment of campylobacteriosis in humans and animals, among the isolates obtained from diverse sources in several countries [5].

Despite the significance of this problem and the major economic and dietary roles of poultry, to the best of our knowledge, no reports were published about the situation regarding campylobacteriosis in poultry or other food products in East Algeria. Therefore, the current study was carried out to determine the prevalence of thermotolerant *Campylobacter* in broiler farms and slaughterhouses located in Batna region (East Algeria) and to investigate the possible effect of the season on the rate of *Campylobacter* infection at broiler chicken farms in this area. The antimicrobial profile of *Campylobacter* isolates obtained in this study was also explored.

## Materials and Methods

### Ethical approval

In this study, we used broiler cloacal swabs, samples from cecum and neck skin of broiler carcasses. Therefore, no ethical approval was needed.

### Sample collection

From June 2016 to June 2018, 960 samples were collected from 6 broiler farms and 12 slaughterhouses randomly chosen in the region of Batna (East Algeria). The poultry farms were located in rural areas and contained broiler houses with a livestock capacity ranging from 4000 to 8000 animals reared in a single band until slaughter. The visited slaughterhouses were located in urban areas, and their processing capacity ranged from 600 to 1200 birds/h. All farms exhibited similar breeding and biosecurity/biosafety protocols.

In terms of the total samples, 480 cloacal swabs were obtained at the farms from broiler flocks 1-2 days prior moving to the slaughterhouse (80 samples from each broiler farm divided into 20 samples for each season), 240 cecal samples were taken after the evisceration of chickens, and 240 fresh broiler chicken neck skin samples were collected at the end of the slaughtering chain. For each slaughterhouse, a single visit was performed early in the morning. Neck skin and cecal content samples were collected from the same slaughter batch and placed in sterile plastic bags and sterile plastic pots, respectively. All samples were placed inside an isothermal cool box at 4°C and transported immediately to the Microbiology Laboratory, University Hospital of Batna, where they were processed within 3-4 h.

To monitor the fluctuations of *Campylobacter* colonization at farms throughout the year, samples from different seasons were included in this study: Summer (June, July, and August), fall (September, October, and November), winter (December, January, and February), and spring (March, April, and May).

### Isolation and identification

The standard protocol of NF ISO 0272-1: 1995 [6] published by the International Organization for Standardization (ISO) and World Organization for Animal Health recommendations [7] were employed for the isolation and identification of thermotolerant *Campylobacter* from all samples. All of *Campylobacter* cultures (enrichment, isolation, identification, and antimicrobial susceptibility testing) were performed under microaerobic conditions (5% O_2_, 10% CO_2_, and 85% N_2_) generated using an anaerobic jar containing a gas generating CampyGen™ reagents (Oxoïd, UK).

For the research on *Campylobacter* from swabs and cecal contents, each sample was plated directly on Karmali medium (Oxoïd, UK) followed by incubation for 48 h at 42°C. For neck skin samples, 10 g of sample was homogenized in 90 mL of Preston’s enrichment broth (Oxoïd, UK) and maintained at 42°C for 24 h before isolation on Karmali agar. Then, the Karmali plates were streaked with one loopful of Preston broth and incubated at 42°C for 48 h in a microaerophilic atmosphere. The plates were checked daily for a total of 5 days for *Campylobacter*, which typically appear as gray, moist flat spreading colonies. If a second incubation was necessary, new generators were used. *C. jejuni* (ATCC^®^ 29428) and *C. fetus* (ATCC^®^ 27374) were used as control strains.

Suspicious colonies were identified by their spreading character and mucoid appearance. Gram-negative bacilli, with spiral morphology and typically high motility with a characteristic corkscrew-like movement, catalase positive, oxidase positive, which did not show growth at 25°C, were presumed to be thermotolerant *Campylobacter* in the preliminary identification.

Presumptive *Campylobacter* colonies were subcultured on Columbia agar (Bio-Rad, France) with 5% horse blood (IPA: Institut Pasteur d’Algérie) and biotyped using a biochemical test on Triple Sugar Iron agar (Oxoïd, UK) of the selective hippurate hydrolysis (Remel, USA) (only *C. jejuni* is hippurate positive). Furthermore, all isolates were tested for their susceptibility to nalidixic acid (30 μg) and cephalothin (30 μg) (Bio-Rad, France) on Mueller-Hinton agar (Oxoïd, UK) with 5% defibrinated horse blood and β-nicotinamide adenine dinucleotide (β-NAD) (MH-F), taking into consideration, the emergence of strains resistant to nalidixic acid.

### Antibiotic sensitivity test

The disk diffusion assay was performed in accordance with the method described by the antibiogram committee of the “French Society of Microbiology” CA-SFM/2014. All isolates were tested for their susceptibility to the following antibiotics: Amoxicillin/clavulanic acid (20/10 μg), ampicillin (10 μg), erythromycin (15 IU), tetracycline (30 IU), gentamicin (10 μg), ciprofloxacin (5 μg), and chloramphenicol (30 μg) (Bio-Rad, France).

From a pure culture of 18-24 h, the bacterial suspension was adjusted to match the 0.5 McFarland turbidity standard. A sterile swab was immersed into the adjusted suspension and then seeded by swabbing onto the entire surface of Mueller-Hinton agar supplemented with 5% defibrinated horse blood and β-NAD (MH-F). After streaking, the inoculum was dried for 5-10 min, and four antimicrobial disks were placed onto the surface of the plate. The plates were incubated at 37°C for 24 h under a microaerophilic atmosphere. Inhibition zones were measured by a caliper, and diameters were interpreted as recommended by the Antibiogram Committee of the French Society of Microbiology [8]. *C. jejuni* ATCC 33560 and *C. coli* ATCC 33876 were used as control strains.

### Statistical analysis

All of the data collected within the present study were analyzed using SPSS 20.0 software (IBM Corp., NY, USA). Chi-squared test (χ^2^ test) was used to compare the prevalence of thermotolerant *Campylobacter* in samples between the farms, the prevalence of isolates according to the sampling season, and the antimicrobial resistance of the isolated strains. Data were considered as significant when p ≤ 0.05 was obtained.

## Results

### Total prevalence of thermotolerant Campylobacter

In this study, thermotolerant *Campylobacter* strains were isolated at high prevalence (63.7%) in all analyzed samples, varying from 65% (312/480) in farms to 62.5% (300/480) in slaughterhouses. More precisely, this bacterium was isolated in 55% (132/240) of neck skin samples, 65% (312/480) of cloacal swabs, and 70% (168/240) of cecal contents (Table-1). The difference between the total prevalence of farms (A,B,C,D,E,F) is not significant (P = 0.16) as shown in Table-2

**Table-1:** Species distribution of *Campylobacter* isolated from broiler farms and slaughterhouses.

Species samples	*Campylobacter jejuni* (%)	*Campylobacter coli* (%)	*Campylobacter lari or Campylobacter coli* resistant to ANC (%)	*Campylobacter upsaliensis* (%)	Prevalence (positive sample/examined samples (%)
Cloacal swabs	250 (80.1)	57 (18.3)	3 (1)	2 (0.6)	312/480 (65)
Ceacal contents	125 (74.4)	37 (22.0)	4 (2.4)	2 (1.2)	168/240 (70)
Neck skin	75 (56.8)	53 (40.1)	3 (2.3)	1 (0.7)	132/240 (55)
Total	450 (73.5)	147 (24.0)	10 (1.6)	5 (0.8)	612//960 (63.7)

**Table-2:** Effect of seasonal variations on the prevalence of thermotolerant *Campylobacter* in broiler farms.

Seasons farms	Summer + (prevalence) (%)	Autumn + (prevalence) (%)	Winter + (prevalence) (%)	Spring + (prevalence) (%)	Total + (prevalence) (%)	p-value (χ^2^ test)
A	20 (100)	16 (80)	4 (20)	12 (60)	52 (65)	0.16
B	20 (100)	15 (75)	12 (60)	10 (50)	57 (71.5)	
C	17 (85)	14 (70)	8 (40)	10 (50)	49 (61.2)	
D	18 (90)	16 (80)	8 (40)	10 (50)	52 (65)	
E	20 (100)	14 (70)	8 (40)	12 (60)	54 (67.5)	
F	18 (90)	14 (70)	6 (30)	10 (50)	48 (60)	
Total	113 (94.2)	89 (74.2)	46 (38.3)	64 (53.3)	312 (65)	
p-value (χ^2^ test)	0.76	0.64	0.18	0.81	
p-value (χ^2^ test)	<0.01					

### Species distribution of thermotolerant Campylobacter isolated from broiler farms and slaughterhouses

Our results showed that, out of the 612 strains identified as belonging to the *Campylobacter* genus, 450 (73.5%) were identified as *C. jejuni*, including 10 resistant and 440 sensitive to ANC, 147 (24%) as *C. coli* (all strains sensitive to ANC), and 5 (0.8%) as *C*. *upsaliensis* sensitive to ANC and cephalothin. The 10 (1.6%) remaining strains were ANC-resistant *C. lari* or *C. coli* (Table-1).

Overall, *C. jejuni* appeared to be more common in cloacal swabs and cecal contents than in neck skin, while *C. coli* was isolated at a higher rate in neck skin than in cecal contents and cloacal swabs. Finally, *C. lari* and *C. upsaliensis* were isolated with a low prevalence in all samples.

### Seasonal variations at farm level

In this study, we also attempted to establish the relationship between the prevalence of thermotolerant *Campylobacter* and the sampling season. Our results showed that *Campylobacter* was more frequently isolated in the summer months (94.2%) than in the other seasons (p<0.05). However, the lowest prevalence was recorded in winter (38.3%). The difference in prevalence among the seasons was highly significant (p<0.001) (Table-2). In addition, the difference in the prevalence of *Campylobacte*r among the six farms in the same season was not significant (*P*_summer_=0.76, *P*_fall_=0.64, *P*_winter_=0.18, and *P*_spring_=0.81) (Table-2).

### Antibiotic susceptibility of isolated strains

All of the strains were tested for their susceptibility to seven antibiotics (Table-3). The results showed that all of the strains were resistant to ampicillin and amoxicillin/clavulanic, while all of them were susceptible to gentamycin. High levels of resistance to erythromycin (83.3%) and tetracycline (66.2%) were also observed. In addition, medium levels of resistance to ciprofloxacin (46.7%) and chloramphenicol (52.6%) were shown.

**Table-3:** Antimicrobial resistance rates of thermotolerant *Campylobacter* isolated strains.

Resistant strain source	Profile	AM	AMC	TE	CIP	E	GM	C
Resistant strain from neck skin	No (%)	132 (100)	132 (100)	88 (66.6)	61 (46.2)	109 (82.6)	0 (00)	69 (52.3)
Resistant strains from cecal contents	No (%)	168 (100)	168 (100)	113 (67.3)	82 (48.8)	142 (84.5)	0 (00)	83 (49.4)
Resistant strains from cloacal swabs	No (%)	312 (100)	312 (100)	204 (65.4)	143 (45.8)	259 (83)	0 (00)	170 (54.5)
Resistant strains total	No (%)	612 (100)	612 (100)	405 (66.2)	286 (46.7)	510 (83.3)	0 (00)	322 (52.6)
p-value		p>0.05	p>0.05	p>0.05	p>0.05	p>0.05	p>0.05	p>0.05

No=Number, AM=Ampicillin, AMC=Amoxicillin/clavulanic acid, C=Chloramphenicol, CIP=Ciprofloxacin, E=Erythromycin, G=Gentamicin, TE=Tetracycline. p-value: Value for the antimicrobial resistance difference between the strains isolated from feces, cecal content and those isolated from neck skin samples to the same antibiotic

No significant differences in resistance to the same antibiotic were observed between the strains isolated from cloacal swabs, cecal contents, and neck skin (p>0.05), but resistance rates among antibiotics used for the same type of sample showed a significant difference (p<0.05). Moreover, all isolates showed multidrug resistance to antibiotics (resistance to two antibiotics or more). More precisely, 0.3% (2/612), 4.9% (30/612), 43.9% (269/612), 47.2% (289/612), and 3.6% (22/612) were resistant to 2, 3, 4, 5, and 6 antibiotics, respectively. Indeed, 16 different resistance profiles were later defined. The results showed that the combination “ampicillin-amoxicillin/clavulanic acid-erythromycin-tetracycline-chloramphenicol” was the most common profile among the campylobacteria, which was identified in 20.7% of the obtained isolates (Table-4).

**Table-4:** Resistance pattern profiles of isolated thermotolerant *Campylobacter* strains.

Associated resistances to	Resistance pattern profiles	Number of strain	Number of total (%)
Two antibiotics	AM, AMC	2	2 (0.3)
	AM, AMC,E	5	
Three antibiotics	AM, AMC, C	5	30 (4.9)
	AM, AMC, CIP	8	
	AM, AMC, TE	12	
	AM, AMC, E, C	35	
	AM, AMC, C, TE	27	
Four antibiotics	AM, AMC, E, TE	113	269 (43.9)
	AM, AMC, E, CIP	75	
	AM, AMC,TE,CIP	12	
	AM, AMC,C,CIP	7	
	AM, AMC, C, E, TE	127	
	AM, AMC, C, E, CIP	70	
Five antibiotics	AM, AMC, C, TE, CIP	29	289 (47.2)
	AM, AMC, TE, CIP, E	63	
Six antibiotics	AM, AMC, C, TE, E, CIP	22	22 (3.6)

AM=Ampicillin, AMC=Amoxicillin/clavulanic acid, C=Chloramphenicol, CIP=Ciprofloxacin, E=Erythromycin, G=Gentamicin, TE=Tetracycline

## Discussion

### Thermotolerant Campylobacter prevalence

In this study, a total number of 960 samples were analyzed for the presence of thermotolerant *Campylobacter*. Bacteriological analysis of samples demonstrated that thermotolerant *Campylobacter* is widespread in East Algeria in both cecal contents and cloacal swabs of broilers, with a concerningly high rate of contaminated carcasses. This confirmed the results of the previous studies in Algeria [9].

### Thermotolerant Campylobacter prevalence in broiler farms

In this study, a prevalence rate of thermotolerant *Campylobacter* of 65% was found among the cloacal swabs sampled from broiler farms. This is similar to the rate of 67.7% reported in North Ireland [10], but higher than 38.1% estimated in South Spain [11]. Conversely, the prevalence rate assessed in the present study is lower than those obtained in several reported studies, varying from 82.9% [12] to 91% [13].

The relatively high prevalence of *Campylobacter* observed in our study can probably be explained by the use of the swabbing technique, which allows scraping of the cloacal mucosa [14], as well as the use of Karmali medium, which shows along with the mCCDA medium the highest yield not only in the recovery of thermotolerant *Campylobacter* but also in the removal of competitive flora [15].

Although little information is available on the prevalence of *Campylobacter* in developing countries, our results are consistent with those reported in Senegal (63%) [16]. However, Gharbi *et al*. and Abushahba *et al*. reported lower prevalence rates of 22.4% and 23.5% in North Tunisia and Upper Egypt, respectively [17,18]. Furthermore, the rates previously reported in Algeria vary from 12% to 96% [19,20].

In this survey, the pathogen was detected at all of the 24 farms, indicating widespread contamination. This finding is supported by the fact that horizontal transmission between birds occurs rapidly (usually within 1-2 weeks), and it is enhanced by fecal excretion and coprophagy of chickens [21].

Finally, it should be noted that the differences in the prevalence of thermotolerant *Campylobacter* found between different studies are likely to be related to a number of factors, such as geographical location [22], sample size, culture methods [23], and age of the subjects [24].

### Thermotolerant Campylobacter prevalence in slaughterhouses

Our study showed a high prevalence of *Campylobacter* in cecal and neck skin samples. Our findings on the prevalence of *Campylobacter* in cecal samples are in agreement with those in a German study in which a similar prevalence (70%) was found [25], but lower than those reported in Algeria (98%) [9] and Grenada (93.5%) [26]. The intestinal carriage is attributed to the adaptation of these enteric bacteria to survival in mucus of the digestive tract [27]. This disparity in the rate of contamination with *Campylobacter* among countries might be due to differences in sampling schemes, analytical methods, and ages of the birds.

We recovered *Campylobacter* from 55% of neck skin samples. This is similar to the prevalence of 60.8% reported by Kovalenko *et al*. in two Latvian broiler chicken slaughterhouses [28]. However, it is higher than the prevalence rates of 24.4% and 27.4% reported in Switzerland and Sri Lanka, respectively [29,30]. In Algeria, our finding is higher than the reported prevalence of 15.7% [31] but lower than the rate of 80% reported by Messad *et al*. [9].

The rates of *Campylobacter* contamination vary widely among countries. In a study by Garin *et al.*, in five major cities located on four continents (Dakar, Yaounde, Noumea, Antananarivo, and Ho Chi Minh City), a range of 15.3%-96.7% was detected, with an average contamination rate of 65.5% [32].

In agreement with Frediani-Wolf *et al*. [28], our study revealed a higher level of *Campylobacter*-positive cecal samples than for the neck skin samples. This finding was further supported by Baré *et al*. [33] who reported that, among the examined chicken parts, neck skin was the most contaminated *by Campylobacter*.

In slaughterhouses, contamination of neck skin could be directly related to ruptured viscera of the same animal and/or to cross-contamination throughout the slaughtering process, perhaps during the evisceration step [34]. Although this evisceration was described as a critical step for *Campylobacter* contamination of carcasses [35], an increase of contamination is not always observed [36].

The nature of slaughter processing makes preventing cross-contamination of negative batches by positive batches impossible [29]. Indeed, the surfaces of carcasses from a *Campylobacter*-free flock were shown to potentially be contaminated when processed after an infected batch of birds due to the pathogen’s ability to survive in water, in aerosols, and on equipment [35]. The traditional character of the visited slaughterhouses with inappropriate sites for evisceration could explain the high rate of contamination in neck skin samples.

### Species distribution

Phenotypic characterization of *Campylobacter* isolates showed that poultry are colonized primarily by *C. jejuni*, followed by *C. coli*, and rarely by other species. These results are in agreement with the previous studies in the Netherlands [37] and Tunisia [17]. However *, C. coli* was isolated at a higher rate than *C. jejuni* in other studies in Egypt [38] and Ecuador [39]. The selective media and incubation temperatures used in this study are more efficient for the isolation of *C. jejuni* and *C. coli* than for other species [40], which raises questions about the validity of our findings on the other strains (*C. lari* and *C*. *upsaliensis*) and indicates the need for further molecular confirmation.

### Seasonal variations at farm level

The prevalence of *Campylobacter* in this study showed seasonal variation. The prevalence was high in summer and fall. Similarly, seasonal variation in *Campylobacter* colonization of broilers with a peak in the warmer periods of the year was reported [41,42]. However, no seasonal variation of *Campylobacter* colonization was found in another study from Great Britain [13].

The definitive reasons for the seasonal variation associated with *Campylobacter* are unknown. However, possible associations between the pathogen’s survival and the temperature [43] and/or the widespread availability of additional reservoirs and mechanical vectors for the disease [44] could explain the high prevalence in summer.

### Antimicrobial resistance of isolated strains

In our study, all tested strains were susceptible to gentamycin. This reinforces the results obtained by Messad *et al*. in Algiers [9]. Similar findings were also reported in Grenada and Canada [25,45]. In fact, this finding was expected since the use of gentamycin in the poultry industry in Algeria was banned since 2006 [46]. Another reasonable explanation for this finding is the fact that gentamicin is rarely used therapeutically because of its intramuscular or subcutaneous route of administration, which is impracticable in the poultry industry [47]. However, in contrast to our finding, a high rate of gentamycin resistance (46.9% of isolates) was reported in another study conducted in the central region of Algeria [20], indicating the possibility that gentamycin has been illegally applied there.

Conversely, a high level of resistance to erythromycin (83.3%) was observed. This is in accordance with the results previously reported in Algeria [20]. This finding is alarming, given that this is considered as one of the first-line antibiotics against human campylobacteriosis. Indeed, during sampling, we observed that tylosin was the antibiotic most commonly used to control respiratory infections in the farms. This could explain the high rate of erythromycin resistance among the isolated *Campylobacter* strains, given that a high frequency of resistance to erythromycin usually occurs in broilers administered tylosin due to cross-resistance [48]. Moreover, Lin *et al*. [49] showed that the use of erythromycin in low doses over a long period (corresponding to its use as a growth factor) selects for resistant strains of *Campylobacter*. In contrast, a low resistance rate of 21.7% was also observed in Algeria [9,19]. In industrialized countries, resistance to erythromycin remains low [26] or even absent [50].

In this study, the rate of resistance to ciprofloxacin was estimated at 46.7%. This is in agreement with the finding by Lutful-Kabir *et al*. in Bangladesh (45.4%) [51] but lower than the rates of 88.1% and 100% reported in China and Latvia, respectively [52,53]. Furthermore, a high level (83.7%) was also reported in Algeria [9]. The high ciprofloxacin resistance rates of *Campylobacter* in Algeria may be attributable to the widespread use of fluoroquinolones in both the prevention and the control of poultry diseases.

The rate of resistance to tetracycline among isolated *Campylobacter* strains was estimated at 66.2%. This agrees with the result of Varga *et al*. [54]. However, a high level of such resistance of 83% was previously identified in Algeria [9,31]. The cause of these high frequencies of resistance in Algeria may be the abuse of tetracycline in broiler farms. Similarly, higher rates of tetracycline resistance were documented in Tunisia [17] and Canada [55]. The authors explained these in terms of tetracycline being used as a growth promoter in the poultry industry.

Our findings regarding the very high levels of resistance to erythromycin and tetracycline (first- and second-line therapeutic agents in human campylobacteriosis) are of great concern and should be emphasized considering that poultry is the major source of human *Campylobacter* infections and antimicrobial-resistant strains can easily be transmitted to humans through the food chain, potentially increasing the campylobacteriosis burden [50].

Although resistance to chloramphenicol is very rare in *Campylobacter* and despite the prohibition of the use of chloramphenicol in Algeria since 2006, the rate of resistance to it in our isolates was estimated at 55.3%. This is very high compared with those reported in the previous studies, in which there was no or only low resistance [9,26,56,57], suggesting that *Campylobacter* generally remains susceptible to this antimicrobial.

The rate of resistance to ampicillin (100%) was relatively consistent with the findings of Guessoum *et al.*, in Algeria, who reported a rate of 81.2% [19]. However, this rate is far higher than the rate of 7% reported in Iran [56]. These high rates of resistance of *Campylobacter* strains to ampicillin are thought to be related to the production of beta-lactamases [58].

The same rate of resistance (100%) was also recorded for amoxicillin/clavulanic acid, which is consistent with the previous study [57]. In contrast, lower rates were previously obtained in Algeria, varying from 27% to 46.8% [9,31].

All of our isolates were multidrug-resistant, which is in agreement with the findings of Messad *et al*. in Algeria [9]. The high prevalence of multidrug-resistant *Campylobacter* in broilers is alarming, given the fact that contaminated poultry meat is the major source of human *Campylobacter* infections.

Foodborne transmission of antibiotic-resistant *Campylobacter* to humans poses a threat to people by limiting the therapeutic choice of antibiotic and compromises the clinical treatment of human campylobacteriosis. Thus, prudent measures for antimicrobial usage and active surveillance should be established to reduce the prevalence and spread of antimicrobial-resistant *Campylobacter*.

## Conclusion

The current study provides the first report about the spread of *Campylobacter* among poultry in East Algeria. Overall, the intestinal carriage and neck skin contamination rates in this study demonstrated high levels of contamination of *Campylobacter* in both broiler farms and slaughterhouses, which peaked in the summer. This study showed that antimicrobial resistance is highly prevalent in the poultry *Campylobacter* isolates, most of which are resistant to multiple antimicrobial agents. An alarming rate of resistance (83.3%) to erythromycin was reported, which is a drug recommended for the treatment of human campylobacteriosis.

Accordingly, the implementation of specific control procedures, monitoring, and preventive approaches such as HACCP from the farm through to the consumer is strongly recommended to reduce the incidence of campylobacteriosis. Therefore, constant monitoring of *Campylobacter* resistance patterns is required, and the use of antibiotics in poultry meat production should be restricted and systematically controlled in Algeria.

## Authors’ Contributions

MB conceived, designed the study, and drafted the manuscript under the supervision of AK and AA. MB and ML designed the experiment protocol under the supervision of AK and HLA. MB collected and analyzed samples. MB, ML, and AH did the statistical analysis. MB and ML revised the manuscript under the supervision of AK and AA. All authors read and approved the final manuscript.
